# ‘Where does my £9000 go?’ Student identities in a marketised British Higher Education Sector

**DOI:** 10.1007/s43545-022-00432-6

**Published:** 2022-07-20

**Authors:** Alice Reynolds

**Affiliations:** Department of Geography, Royal Holloway, University of London, Egham, UK

**Keywords:** Entitlement, Consumers, Identity, Instrumentalism, Marketisation, Students

## Abstract

Significant evidence highlights processes of marketisation within British higher education since the 1980s, with changes to the funding, management, and expectations of higher education institutions, students, and staff. Through a cross-national and cross-institutional analysis, this paper explores the identities of students within a marketised British higher education landscape, and specifically, explores the identity of the ‘student-consumer’. Using a mixed methods approach with students from 37 higher education institutions across Britain, this research explores the attitudes, expectations, behaviours, and relationships held by students regarding higher education. Student identity orientations are explored, before the extent to which students’ express attitudes of instrumentalism and entitlement is investigated. The paper concludes that whilst there is evidence of consumerist discourses framing students’ relationship to higher education, students challenge the perception that they are passive consumers, and instead recognise the need to remain active co-producers throughout higher education. These findings have implications for policy and have resonance beyond Britain, as the marketisation of higher education is an increasingly international phenomenon.

## Introduction

This paper explores the identities of students within a marketised British higher education (HE) landscape, and specifically, explores the identity of the ‘student-consumer’—a term which has come to dominate HE policy and media discourse. The British HE landscape has dramatically transformed over the last 30 years, characterised by “significant and rapid change” (Foskett 2010, p. 25). British HE has been subject to marketisation since the 1980s, accompanied by reforms shifting HE in a corporate direction (Brown 2015). Brown (2011, p. 1) defines marketisation as: ‘the application of the economic theory of the market to the provision of higher education’. Jongbloed (2003, p. 113) further indicates that the marketisation of HE is ‘aimed at strengthening student choice and liberating markets in order to increase quality and variety of services offered by the providers of higher education’.

Notions of marketisation and competition have grown in national policy rhetoric in the UK. Since the UK first identified students as customers within the 1997 Dearing report (Dearing 1997), Higher Education Institutions (HEIs) have become increasingly commercialised. More recently, the HE White Paper, *Higher Education: Success as a Knowledge Economy*, and the *Higher Education and Research Act 2017* in the UK promotes the creation of a ‘competitive market’ as a principle for HE reform (Government of the United Kingdom 2017). Tuition fees have been introduced and increased, student grants have been replaced by loans, rankings and league tables guiding student choice are proliferating, and higher education institutions are dedicating increased resources to marketing, branding and customer service (Brown 2011).

It is important to note that HE policy differs across England, Scotland, and Wales, most significantly in terms of tuition fees. HE tuition fees were first introduced across Britain in 1998 after the publication of the Dearing report a year earlier, with students required to pay up to £1000 per year for tuition. However, different fee arrangements now exist in each of the devolved administrations. Full-time undergraduate UK students are required to pay up to £9250 in England and £9000 per year in Wales for tuition fees, with access to government student loans through the Student Loans Company (Student Finance England and Student Finance Wales). For Scottish students attending universities in Scotland, most universities charge up to £1820 per year for undergraduate tuition fees (University of Edinburgh 2022). However, most Scottish students are eligible to have their fees paid by the Scottish government via the Student Awards Agency for Scotland (SAAS), meaning effectively, they do not have to pay tuition fees when studying in Scotland. Students from England and Wales can be charged up to £9250 per year to study north of the border and cannot apply to the SAAS. Scottish students who choose to study outside of Scotland and elsewhere in Britain will be charged the tuition fees set by their chosen institution but may still apply for a loan to cover the costs through SAAS. Prior to Brexit, EU students were able to study at UK institutions as ‘home students’ and therefore avail of lower fees and access to student loans. However, those starting a course in 2021/22 no longer have home fee status or access to the UK’s financial support, therefore having to pay international student fees, which are considerably higher. This excludes Irish students, who are covered by the Common Travel Area. International undergraduate tuition fees vary considerably at British universities, starting at around £10,000 per year, but in many cases is considerably more. Postgraduate tuition fees also vary for both domestic and international students, ranging from around £4900 to £30,000 a year, with the average around £11,000 a year (UCAS 2022).

Debates concerning the rise of student consumerism—an ‘attitude that treats the university as a place to meet pre-established needs’ (Delucchi and Korgen 2002, p. 101)—are proliferating. This is in part due to the tripling of domestic student undergraduate fees in England in 2012, with HEIs now permitted to charge £9250 per annum to British students, accompanied by the announcement that HE students ‘who have purchased a service (the provision of education)’ (Mawji, 2016) are protected under the Consumer Rights Act (2015). Despite differences in fee regimes between the devolved nations, HE in Scotland and Wales is not unaffected by political developments at the British level, and marketisation trends dominate policy across all three jurisdictions. For example, the research excellence framework (REF), first conducted in 2014, is a UK wide system for assessing the quality of research carried out in publicly-funded universities and increases competition between universities by being used to inform the selective allocation of research funding to HEIs. The UK Higher Education and Research Act 2017 further promotes consumer relations between universities and students. Furthermore, ‘the student experience’ has become a buzzword within HE policy. The National Student Survey (NSS) in the UK, for example, gathers students’ opinions on the quality of their courses and makes the results publicly available to ‘inform prospective students’ choices; provide data that supports universities and colleges to improve the student experience; and support public accountability’ (OfS, 2022). Marketisation trends are therefore UK wide. Policy drives have ‘stressed higher education as a private rather than a public good, the role of students as rationally calculating consumers and a conception of HEIs as competing businesses’ (Telling, 2018, p. 1294).

A central aim of this article is to investigate how students understand their identity within a marketised HE sector. This is important given the increasing assumptions that are made about common understandings of ‘the student’ within HE policy and media discourse, which commonly positions students as ‘consumers’. Despite the pervasiveness of the conceptualisation of the student-consumer in media and political discourse, discussion has only recently emerged on the extent to which HE students themselves perceive their identity. Whether students identify as, behave like, or navigate through HE as a ‘consumer’ remains a ‘contested yet under-analysed entity’ (Nixon et al. 2016, p. 1). As Brooks et al. (2021) assert, claims that students have become consumer-like in their behaviour are often made based on limited empirical evidence, rather than exploring the perspective of students themselves. This research therefore helps to address this gap in the literature and contributes to a growing body of empirical work to emerge over the last 5 years which engages with the changing identities of students in a marketised British higher education sector (Tomlinson 2017; Budd 2016; Bunce et al. 2016). As such, the following research questions were formulated to guide this research:

1. To what extent do students at British HEIs express a consumer orientation towards HE?
2. To what extent has the marketisation of British HE led to attitudes of instrumentalism?
3. To what extent has the marketisation of British HE led to attitudes of student entitlement?

Conceptually, this research is underpinned by the argument that the marketisation of HE has led to the identity of students as consumers. Given the growing, but limited, extant empirical research which places students at the heart of the research, this paper explores the attitudes, expectations, behaviours, and relationships held by students regarding HE. Saunders (2015) argues for the need to provide a reliable understanding of student identities. This paper begins with a review of existing literature. As the marketisation of UK HE has been documented in detail elsewhere, this review will focus on reviewing the literature relevant to the central themes of this paper, whilst acknowledging the wider HE marketisation debates. In the section which follows, data exploring student identities will be presented and discussed. The paper will then draw upon the empirical research findings to investigate two central impacts of the marketisation of HE to emerge from existing literature: instrumental attitudes to learning, and attitudes of entitlement. Finally, concluding thoughts and avenues for future research are presented.

## Changing student identities

The student-consumer has increasingly captured the attention of scholars internationally (Budd 2016; Bunce et al. 2016; Molesworth et al. 2009; Naidoo et al. 2011; Tomlinson 2017; Bossick 2009; Delucchi and Korgen 2002; Clayson and Hayley 2005; Obermiller et al. 2005; Cuthbert 2010; Tavares and Cardoso 2013; Silverio et al. 2021; Jayadeva et al. 2021; Brooks and Abrahams 2020). Whilst it has been argued that a consumer identity is increasingly recognised and internalised by students (Bunce et al. 2016), empirical evidence to support the extent to which students express or perform a consumer identity remains limited (Naidoo and Jamieson 2005; Saunders 2015). Lomas (2007) explores whether students are consumers by interviewing academic staff but omits the perceptions of students themselves. Similarly, von Alberti-Alhtaybat et al. (2017) compare the effects of HE policies in England and Germany but interview accounting professors only. Recent work has also explored the identity of students as consumers from the perspective of students’ union leaders across Europe (Brooks 2021). Furthermore, McCollough and Gremler (1999) explore the consequences of treating students as customers, but in doing so assume students view themselves as customers without demur.

This paper advances recent research which explores how students themselves understand and experience HE. Budd (2016) explores undergraduate orientations towards HE in Germany and England in the spring and summer of 2012, just prior to the tripling of tuition fees in England. In addition, Tomlinson (2017) explores student perceptions of themselves as ‘consumers’ of higher education by interviewing students who had entered HE both before and after the tripling of tuition fees in England, enabling the research to explore the impacts of higher tuition fees on student identities.

Existing research on student identities focuses largely on US college students. Given the influence of American HE upon the UK (Williams 2013), examining such research is significant. A study by Saunders (2015) on first-year students at one university in the US found that only 28.9% of respondents expressed a customer orientation. A further study suggested two contrasting orientations: ‘students as customers’ and ‘students as products’ (Obermiller et al. 2005). Whilst the results suggest that students perform a consumer as opposed to a product orientation, it is not acknowledged that students can be more than ‘customers’ or ‘products’ (Saunders, 2015).

Critiques of the student-consumer as the dominant identity of HE students (Nixon et al. 2016) sparks debates concerning students as active customers (Barnett 2011); citizens (Svensson and Wood 2007); members, people (Cuthbert 2010); judge, partners (Clayson and Hayley, 2005); choosers (Nixon et al. 2016); apprentices (Saunders 2015); trainees, clients or employees (Finney and Finney 2010); policy pawn (Tight 2013); learners (Bunce et al. 2016; Cuthbert 2010), co-producers (McCulloch 2009); co-creators (Kalafatis and Ledden 2013; Naidoo et al. 2011; Ng and Forbes 2009); change agents (Zandstra and Dunne 2009), prosumers (Sharif 2014) and counter-hegemonic producers (Neary and Winn 2009). The student-consumer therefore remains ‘one possible position amongst many’ (Nixon et al. 2016, p. 3). This paper therefore explores the identity of students beyond a consumer orientation.

## Instrumentalism

Students are increasingly positioned as taking an instrumental approach to HE (Muddiman 2018). In positioning students as consumers, Molesworth et al. (2009) argue this generates an attitude whereby students seek to ‘have’ a degree rather than ‘be’ scholars willing to engage intellectually, resulting in passive instrumental attitudes to learning. Budd (2016, p. 25) argues paying tuition fees results in university degrees becoming an ‘investment in the self’, whereby students make decisions which will benefit their future employment and income opportunities (Marginson 2006). Kaye et al. (2006) support this argument, stating students ‘want to see obvious, tangible benefits from their studies, whether in terms of inherently valuable qualifications or as a route to a particular form of employment’. Fromm’s theory based on ‘having’ is of particular significance within HE discourse, associating pedagogic philosophy to a critique of consumer culture which creates a society based on ‘having’ (Molesworth et al. 2009). Fromm argues the student-consumer longs to ‘have’ a degree as if it were a commodity, and in doing so ‘train[s] people to have knowledge as a possession’ (Fromm 1976, p. 34).

Fromm attributes pedagogic theory to a critique of consumer culture, inferring that education cannot be ‘had’ but ‘experienced’ (Molesworth et al. 2009). However, absent from this argument is the idea that students can both ‘have’ and ‘be’ at the same time. Many students are enthusiastic about their subject of study and seek to be intellectually challenged (Molesworth et al. 2009), thus remain critical about accusations they have ‘bought’ their degree. Shankar and Fitchett (2002) note the structural dualism, suggesting students can both be in a position of ‘having’, at the same time as achieving satisfaction through a state of ‘being’.

Despite increased attention towards instrumentalism within HE, empirical examination remains scarce (Muddiman 2018). Those which have offer a critique of Fromm’s theory. Hemsley-Brown and Oplatka (2015) found over 40 ‘choice actors’ regarding students’ university selection and found only a small selection of which were employment or financially orientated. Davies et al. (2013) found that men and certain ethnic groups were more likely to attend university based on labour market and income factors, but they and others also cited factors including job status, creativity and altruism as influencing HE decisions. Furthermore, Muddiman (2018) found students studying business presented elements of instrumental or ‘having’ attitudes to learning, whilst those studying sociology presented less instrumental ‘being’ modes of learning.

## Entitlement

As the marketisation of HE intensifies, so has the debate regarding student entitlement (SE), which ‘indicates that on some level students believe they are entitled to or deserving of certain goods and services to be provided by their institutions and professors, something that is outside of the student’s actual performance or responsibilities inside the classroom’ (Singleton-Jackson et al. 2010, p. 344). Interpreting this definition, students feel ‘entitled’ or ‘deserving’ of a degree despite whether they have demonstrated the academic performance to do so. Williams (2013, p. 6) positions the student-consumer as ‘someone who, as a result of financial exchange, considers themselves to have purchased, and is therefore entitled to possess, a particular product (a degree) or to expect access to a certain level of service (staff and resources)’. In addition, King (1993) and Sacks (1996) draw attention to a growing culture of entitlement whereby students not only feel entitled to a degree but perceive achieving top grades as a ‘right’.

Morrow (1994) was one of the first to write about SE and how it relates to achievements in education, positioning it as a cultural shift ‘away from the values of education as they pertain to learning and towards an award and achievement approach that undermines the nature of scholarship’ (Singleton-Jackson et al. 2010, p. 344). Morrow (1994) outlined examples of the immorality of achievement, including a rise of cheating, plagiarism, and the purchasing of degrees. Feelings of entitlement, Singleton-Jackson et al. (2010, p. 345) argue, ‘defeats academic achievement by denying the significance of learning to the learner’.

Following Morrow emerged a growth in attention towards SE. Greenberger et al. (2008) found that media references for the combined search terms of ‘sense of entitlement’ and ‘students’ increased from 16 in 1996 to 102 in 2006 in the Lexis/Nexis online journal database. However, they further noted that SE had yet to be examined in depth by scholars. Existing literature provides a variety of findings and explanations regarding origins of feelings of entitlement (Singleton-Jackson et al. 2010). Some researchers have explored the extent to which SE results from a self-centred, narcissistic approach to education (Foster et al. 2003; Hoover 2007). Ciani et al. (2008) further explored the understanding of SE, finding that the longer students are within HE the more entitled they feel, with males feeling a higher sense of entitlement overall. Further research finds that ‘customer’ orientations are highest amongst business, arts and science students (Obermiller et al. 2005; Delucchi and Korgen 2002). Such observations highlight the need for further research into attitudes of SE within HE, with specific examination of the influence of individual variables.

## Methodology

The aim of this paper is to provide a cross-national and cross-institutional analysis of student identities within a marketised British HE landscape. To achieve this, the paper comprises of mixed methods research conducted with HE students and alumni from 37 British HEIs in the spring and summer of 2017. The research consisted of a two-stage research design. 101 online questionnaire responses were collected, followed by 10 semi-structured interviews. Given the impact of tuition fees on the marketisation of HE, research was conducted with students who had attended HE post September 1998, when tuition fees were first introduced in the UK. Universities represented include Russel Group, Red Brick, and new universities/polytechnics, representing the diversity of British HEIs. Participants were the ages of 17–45 + , with the largest sample, 56.44% (*n* = 57), aged 21–25. The smallest sample, 0.99% (*n* = 1), were aged over 45. 75.27% (*n* = 74) of respondents were female and 26.74% (*n* = 27) were male. 91.09% (*n* = 92) of respondents were home students, 6.93% (*n* = 7) were from the EU, and 1.98% (*n* = 2) were international students. 85.15% (*n* = 86) of participants funded HE through a government student loan and 22.77% (*n* = 23) funded HE through family support.

Before commencing, the research received clearance from a university ethics committee. The first stage of the research design, the online questionnaire, relied on passive recruitment methods and was distributed via social media on Facebook in student member groups and on student forums on The Student Room. This allowed the questionnaire to reach a diverse group of students and alumni. Snowballing assisted in further recruitment, providing an effective way of collecting data from a diverse sample. Answers remained anonymous unless participants chose to leave their details to be contacted for stage two of the research design. The questionnaire consisted of 12 questions presented to respondents in a predetermined order, providing an efficient way of collecting data from a large sample prior to analysis (Saunders et al. 2012). Questions were both open and closed and therefore presented mixed methods results. Question 9 of the questionnaire asked respondents the extent to which they agreed with 18 statements concerning their education and identity on a five-part Likert scale (Table 1). Statements were adapted and incorporated from Saunders (2015) and Bunce et al. (2016), in addition to the incorporation of original statements. Question 10 asked participants to what extent they perceived themselves to be a range of identity labels (Table 2). To ensure the appropriateness of the chosen statements, statements underwent preliminary testing, and the pre-test questionnaire was answered by 15 participants. Due to testing, some statements were removed, and others were re-worded to improve clarity. The distribution of an online questionnaire provided access to a diverse group of participants quickly and at a low cost (Wright 2005).

**Table 1:** Student identity orientation response distributions (*n* = 101) (statements adapted from Saunders (2015) and Bunce et al. (2016))

	Agree strongly (1)	Agree somewhat (2)	Neither agree nor disagree (3)	Disagree somewhat (4)	Disagree strongly (5)
I think of university education as a product	11.88% (*n* = 12)	47.52% (*n* = 48)	14.85% (*n* = 15)	18.81% (*n* = 19)	6.93% (*n* = 7)
I chose/choose modules based on whether they will help my future career	28.71% (*n* = 29)	35.64% (*n* = 36)	9.90% (*n* = 10)	17.82% (*n* = 18)	7.92% (*n* = 8)
I think of myself as a customer of my university	10.89% (*n* = 11)	33.66% (*n* = 37)	8.91% (*n* = 9)	33.66% (*n* = 34)	12.87% (*n* = 13)
I chose to attend university because I enjoy studying	40.59% (*n* = 41)	36.63% (*n* = 37)	11.88% (*n* = 12)	10.89% (*n* = 11)	0.00% (*n* = 0)
I chose to attend university because I didn’t know what else to do	8.91% (*n* = 9)	28.71% (*n* = 29)	5.94% (*n* = 6)	18.81% (*n* = 19)	37.62% (*n* = 38)
If I complete all my assignments, I deserve a good grade no matter what	0.99% (*n* = 1)	9.90% (*n* = 10)	10.89% (*n* = 11)	39.60% (*n* = 40)	38.61% (*n* = 39)
I find it more important to get a good grade than to learn the course content	8.91% (*n* = 9)	31.68% (*n* = 32)	23.76% (*n* = 24)	28.71% (*n* = 29)	6.93% (*n* = *7)*
I chose my degree based on how much money I can make in the future	0.99% (*n* = 1)	22.77% (*n* = 23)	6.93% (*n* = 7)	29.70% (*n* = 30)	39.60% (*n* = 49)
Education is something I receive, not create	1.98% (*n* = 2)	42.57% (*n* = 43)	23.76% (*n* = 24)	28.71% (*n* = 29)	2.97% (*n* = 3)
Education is a service	11.88% (*n* = 12)	61.39% (*n* = 62)	11.88% (*n* = 12)	13.86% (*n* = 14)	0.99% (*n* = 1)
Developing critical thinking is only a positive if it helps in my future career	1.98% (*n* = 2)	14.85% (*n* = 15)	12.87% (*n* = 13)	44.55% (*n* = 45)	25.74% (*n* = 26)
My grade should always be bumped up if I’m close to an upper grade boundary	8.91% (*n* = 9)	20.79% (*n* = 21)	29.70% (*n* = 30)	20.79% (*n* = 21)	19.80% (*n* = 20)
My attendance and effort (i.e. attending class and doing readings) should be part of my university grades	16.83% (*n* = 17)	38.61% (*n* = 39)	13.86% (*n* = 14)	20.79% (*n* = 21)	9.90% (*n* = 10)
I chose my modules based on how easy they were	0.99% (*n* = 1)	19.80% (*n* = 20)	9.90% (*n* = 10)	31.68% (*n* = 32)	37.62% (*n* = 38)
I perceive myself as a co-producer of my education	29.70% (*n* = 30)	44.55% (*n* = 45)	13.86% (*n* = 14)	9.90% (*n* = 10)	1.98% (*n* = 2)
I chose my modules based on how much work they involved	1.98% (*n* = 2)	36.63% (*n* = 37)	10.89% (*n* = 11)	29.70% (*n* = 30)	20.79% (*n* = 21)
Because I paid for my education, I should have more say over what is taught and how	19.80% (*n* = 20)	45.54% (*n* = 46)	13.86% (*n* = 14)	16.83% (*n* = 17)	3.96% (*n* = 4)
Because I paid to attend university, I deserve a degree	7.92% (*n* = 8)	9.90% (*n* = 10)	5.94% (*n* = 6)	38.61% (*n* = 39)	37.62% (*n* = 38)

**Table 2:** Student identity label response distributions (*n* = 101)

	Agree strongly (1)	Agree somewhat (2)	Neither agree nor disagree (3)	Disagree somewhat (4)	Disagree strongly (5)
Student	84.16% (*n* = 85)	10.89% (*n* = 11)	2.97% (*n* = 3)	1.98% (*n* = 2)	0.00% (*n* = 0)
Consumer	5.94% (*n* = 6)	37.62% (*n* = 38)	14.85% (*n* = 15)	28.71% (*n* = 29)	12.87% (*n* = 13)
Prosumer	6.93% (*n* = 7)	11.88% (*n* = 12)	62.38% (*n* = 63)	11.88% (*n* = 12)	6.93% (*n* = 7)
Producer	5.94% (*n* = 6)	28.71% (*n* = 29)	32.67% (*n* = 33)	23.76% (*n* = 24)	8.91% (*n* = 9)
Customer	9.90% (*n* = 10)	37.62% (*n* = 38)	17.82% (*n* = 18)	19.80% (*n* = 20)	14.85% (*n* = 15)
Learner	76.24% (*n* = 77)	22.77% (*n* = 23)	0.99% (*n* = 1)	0.00% (*n* = 0)	0.00% (*n* = 0)
Stakeholder	15.84% (*n* = 16)	22.77% (*n* = 23)	0.99% (*n* = 23)	0.00% (*n* = 23)	0.00% (*n* = 16)
Member	36.63% (*n* = 37)	50.50% (*n* = 51)	4.95% (*n* = 5)	5.94% (*n* = 6)	1.98% (*n* = 2)
Citizen	31.68% (*n* = 32)	37.62% (*n* = 38)	20.79% (*n* = 21)	34.65% (*n* = 7)	14.85% (*n* = 3)
Client	6.93% (*n* = 7)	22.77% (*n* = 23)	20.79% (*n* = 21)	34.65% (*n* = 35)	14.85% (*n* = 15)
Employer	0.99% (*n* = 1)	1.98% (*n* = 2)	14.85% (*n* = 15)	31.68% (*n* = 32)	50.50% (*n* = 51)
Co-producer	5.94% (*n* = 6)	27.72% (*n* = 28)	35.64% (*n* = 36)	18.81% (*n* = 19)	11.88% (*n* = 12)
Counter-hegemonic producer	0.00% (*n* = 0)	5.94% (*n* = 6)	69.31% (*n* = 70)	12.87% (*n* = 13)	11.88% (*n* = 12)

The second stage of the research design consisted of semi-structured interviews, which assisted in explaining the data collected in the first stage of the research by exploring participants’ views in greater depth. Participants were recruited through the completion of the online questionnaire. Respondents were asked if they would be happy to be contacted for further research, and if yes, were asked to leave an email address. This proved a successful recruitment method as 32 respondents (31.68%) agreed to be contacted further. A purposive sampling strategy—maximum variation sampling—was selected to allow for the ‘maximum variation possible in the data collected’ (Saunders et al. 2012, p. 301). Participants were selected from different institutions, academic departments, were at different stages of HE, and had funded HE in different ways (information displayed after each quote presented in this paper). Interview participants provided written informed consent before participating. Students were asked overt ‘consumer based’ questions to explore the range of responses given when approached this way (Singleton-Jackson et al. 2010), in addition to more general questions about their opinions and attitudes towards HE. Thematic areas covered included: reasons for attending university or studying chosen subject; attitudes towards tuition fees; the role of HEIs; relationships between students and staff; and more general views towards the marketisation of HE. Whilst a set of predetermined questions were prepared, respondents were encouraged to interpret the questions and freely discuss the topic at hand (e.g. ‘What do you believe you should get out of university?’) Interviews allowed for in-depth, exploratory research, assisting in consolidating the data collected in the first phase of the research design.

Exploratory data analysis of the quantitative questionnaire results occurred, with summary statistics presented in tables and graphical displays allowing for visual analysis of the results. Interviews were audio recorded and later transcribed verbatim. Thematic analysis, a ‘foundational method for qualitative analysis’ (Braun and Clarke 2006, p. 78) allowed for the identification, analysis and reporting of patterns within the qualitative questionnaire and interview data. A two-stage coding cycle was implemented. The first stage involved holistic coding as an exploratory coding method, complimented by open coding as an elemental coding method. This created a sense of the overall contents of the data (Saldaña 2016). This led to the second coding cycle, during which focused coding combined the codes into higher order categories (Saldaña 2016). For example, students’ comments stating education was their ‘right’ were designated in the ‘Student Entitlement’ theme. Qualitative data collected from the questionnaire and interviews were merged into a single, cohesive dataset, with coded responses situated into key themes (Singleton-Jackson et al. 2010).

## Findings and discussion

### Student identity positions

To understand student identities within a marketised HE sector, questionnaire respondents were asked to what extent they agreed with thirteen identity orientations (Table 2). The identity which respondents agreed with most strongly, perhaps obviously, was ‘Student’—84.16% (*n* = 85) ‘agreed strongly’ that they associate with the identity of ‘student’. Respondents also associated with ‘Learner’—74.24% (*n* = 77) ‘agreed strongly’. Yet nearly a quarter of respondents (22.77% (*n* = 23) only ‘agreed somewhat’ that they are a ‘Learner’, suggesting it is not the only identity with which these respondents associate. Congruent with Saunders (2015, p. 25), therefore, students ‘do not have a monolithic identity’ and cannot be reduced to one dimension (Cuthbert 2010).

Respondents also associated themselves with the identity of a ‘citizen’—31.86% (*n* = 32) ‘agreed strongly’ and 37.62% (*n* = 38) ‘agreed somewhat’, and ‘member’—33.63% (*n* = 37) ‘agreed strongly’ and 50.50% (*n* = 51) ‘agreed somewhat’ that they were ‘members’ of their HEI:

- ‘I think we’re more like members. Because when something goes wrong on Amazon for example, you contact customer support if something goes wrong. For students, we contact lecturers when we need support. Lecturers are like customer support. You expect them to respond in good time’. (Participant E, Male, Graduate, Psychology, 11 contact hours a week)

These findings support Cuthbert (2010, p. 15) who states that students are members of a university, or ‘“citizens” in a kind of academic democracy’, with right to the participation in university governance. According to Hirschman (1970), members of any organisation have three options—exit, voice, or loyalty. If HEIs do not want their students, or ‘members’, to leave, it must support them in voicing their concerns, and build upon their loyalty to their institution (Cuthbert, 2010). Whilst Participant E states he perceives himself as a ‘member’ rather than a consumer, he demonstrates consumer behaviour by perceiving lecturers as providing ‘customer support’.

Whilst only 5.94% (*n* = 6) of questionnaire respondents ‘agreed strongly’ that they were consumers of HE, 19.80% (*n* = 20) ‘agreed strongly’ that they ‘should have more say over what is taught and how’. These findings suggest the presence of a contradictory consciousness within students (Saunders, 2015). This occurs when students convey views that are congruent with the dominant ideology yet discard the exact manifestations of the ideology within their own lives (Cheal, 1979; Gramsci, 1971). Therefore, students may not directly sense the contradiction between their responses (for example, by stating they are a member rather than a consumer but expecting ‘customer support’). We must therefore remain cautious when drawing conclusions from the analysis of single-item responses (Saunders, 2015). The presence of contradictory consciousness highlights the need for further investigation as the marketisation of the sector intensifies.

Many scholars agree that as public financial support decreases and students are faced with rising tuition fees, HE is ‘increasingly embedded in the discursive realm of consumerism’ (Schwartzman, 2013, p. 43). Supporting Bunce et al. (2016) who found that a higher fee responsibility is associated with a higher consumer orientation, the findings in this paper confirm a direct correlation between the payment of tuition fees and student’s expressing consumerist identities:

- ‘Do you see yourself as a consumer or customer of your university?’
  - ‘Yes definitely. I’ve chosen to go here and I’ve chosen to buy into the system. So yes, I would say I’m a customer, but I still have to put the work in. It’s a bit more of a grey area than that’. (Participant C, Female, UG, Drama, 6 contact hours a week).
  - ‘Definitely. They sold it to us and they treat and respect us like customers. The student satisfaction surveys are exactly the same as a customer satisfaction survey’. (Participant J, Male, PG, Earth Sciences, 20 contact hours a week.)
  - ‘Not necess… Yes. Yes I am actually. I am in a sense, because if I didn’t pay my fees, I wouldn’t be able to go to university. So I am a consumer, but I only really feel like that when the fees come around, so it’s a temporary feeling. You are a customer of a university because you have to pay the money to attend’. (Participant I, Male, UG, Mathematics, 16 contact hours a week)

This research found a stronger consumer identity was held by those who made a direct financial exchange. When asked whether she perceived her degree as a product, one postgraduate student said:

- ‘Not really with my undergrad degree, but I think more so with my masters. I think because with my undergrad tuition the money never came into my bank account, so I never really thought about the fact that I paid £27,000 to go to university. But in doing a masters, the money comes into my bank and then I have to pay it each term, so I see it more as a product this year as I’ve physically seen the money go in and out’. (Participant A, Female, 22, PG, Geography, 4 contact hours a week)

Whilst attitudes of being a ‘consumer’ were highlighted within multiple interviews, for many, a consumer identity was not the primary identity held by students but was one which they understood or related to due to the payment of tuition fees. Those never in direct possession of their tuition fee funds (such as undergraduate students with a student loan), perceive their education less as a product (and therefore expressed less of a consumer identity) than those who made a direct financial exchange (such as Participant A). This supports the assertion that students should not only, if at all, be seen as consumers, but in other significant ways: as learners, members, citizens, employees, clients and as people (Cuthbert, 2010). Arguably, by positioning students solely as consumers, this oversimplifies their roles and activities within HE.

### Instrumentalism

Students are increasingly positioned as ‘commodity purchasers’ and lecturers as ‘information brokers’, whose role it is to package and present information efficiently (Tomlinson 2017, p. 454). This has led to hypotheses that students are consumers of a HE ‘product’ (Morrison 2016), as opposed to a ‘process’ (Tomlinson 2017). When presented with the statement: ‘I think of my university education as a product I am purchasing’, 11.88% (*n* = 12) respondents ‘agreed strongly’ and 47.52% (*n* = 48) ‘agreed somewhat’. Others perceived neither education nor a degree as a product, but they themselves as a product:

- ‘I see myself more as a product, and a degree as something that is contributing to me. It’s a contribution to me as a product and I’m selling myself [on the labour market]. I don’t think the degree itself is a product’. (Participant H, Female, PG, Management/Law, 6 contact hours a week)

Rather than focusing on the ‘use value’ of the knowledge gained through HE, students are recognised as focusing on the ‘exchange value’ of their degree through the transformation into economic capital (Labaree 1997). The rise of buying and selling degree certificates online is a key example of this consumerist turn. Students have long been under pressure to maintain drive for self-development and intellectual growth, at the same time as ‘having’ the set requirements and knowledge to operate in the labour market (Molesworth et al. 2009). The latter instrumental approach supports the findings from this research of students’ motivations to study at HE level. The research found an overriding trend of students attending university for the purposes of enhancing employment prospects, with 72.27% (*n* = 73) of questionnaire respondents making specific reference to advancing employment opportunities, salaries and future careers:

- ‘Further educational experience for my career path’
- ‘To be of a degree-level standard, for better paid work’
- ‘Usual narrative of “opens more employment doors”’

28.71% (*n* = 29) of questionnaire respondents ‘agreed strongly’ and 35.64% (*n* = 36) ‘agreed somewhat’ that they ‘chose/choose modules based on whether they will help [their] future career’. These findings are concurrent with Brooks et al. (2021) whose research with students found that the most common purpose of HE was to prepare themselves for the labour market. This supports the view of student consumerism which positions the student-consumer as focused on gaining access to a financially rewarding career (Bossick 2009).

Whilst participants agreed that ‘having’ a degree was a device to enhance their futures, this was not at the expense of their willingness to engage intellectually. 44.55% (*n* = 45) participants ‘disagree[d] somewhat’ and 25.74% (*n* = 26) ‘disagree[d] strongly’ with the following statement: ‘Developing critical thinking is only a positive if it helps my future career’. They further disagreed that they selected modules based on how easy they were—31.68% (*n* = 32) ‘disagree[d] somewhat’ and 37.62% (*n* = 38) ‘disagree[d] strongly’. In addition to desires to improve employment prospects, students expressed longing to learn and engage with intellectual content:

- ‘I genuinely enjoy studying and wanted to learn a new language and continue studying new areas of literature. I’m also aware that a degree is necessary for many of the career paths that I’m interested in’. (Questionnaire respondent)
- ‘To improve employability and engage in further research into my chosen subject’. (Questionnaire respondent)
- ‘I think university is more than just getting a degree, it’s also about learning life skills’. (Participant A, PG, Geography, 4 contact hours a week)

This critiques Fromm’s (1976) theory based on having, which positions students as a group who seek to ‘have’ a degree at the expense of ‘being’ scholars. Whilst Williams (2013, p. 8) draws attention to cultural assumptions of students who ‘seek passive possession of a degree commodity rather than active intellectual engagement of the learning process’, students recognised they ‘still have to put the work in’ (Participant C) and shouldn’t ‘be handed something just because you’ve paid for it’ (Participant H). This supports Shankar and Fitchett (2002) who suggest that students can both be in a position of having a degree, at the same time as achieving satisfaction through being a learner. This questions the view that the student-consumer has become the dominant identity of HE students, in turn harming pedagogic relations (Tomlinson 2017). Whilst contemporary students seek to ‘have’ a degree to compete in the labour market, they equally wish to ‘learn’, ‘improve’ and ‘engage’ (quotes from questionnaire respondents), seeking both to ‘have’ and to ‘be’ (Shankar and Fitchett 2002).

### Entitlement

In contrast to literature which suggests students perceive achieving a degree as their ‘right’, when asked whether they agree with the following statement—‘Because I paid to attend university, I deserve a degree’, 76.23% (*n* = 77) questionnaire respondents disagreed. Respondents asserted they should not be awarded for poor effort and did not believe they should be guaranteed a degree due to the payment of tuition fees.

- ‘Do you think you should be guaranteed a degree through the payment of tuition fees?’
  - ‘No, purely because of the people who pay the same amount of money, but don’t attend lectures and don’t put the effort in. Why should they get the same output as someone who tried their best and puts their all in? I think you have to work for it, and if you’re not willing to work for it then it says a lot about you. I don’t think you should be handed something just because you’ve paid for it’. (Participant H, Female, 22, PG, Management/Law, 6 contact hours a week)

This suggests students recognise their identity as co-producers in their educational outcomes. Positioning students as co-producers highlight the *process* of the learning experience over the end *product* (Lane 2017). This supports Brooks and Abrahams (2020) who find students typically foregrounded learning and hard work rather than more instrumental concerns which are typically implied within policy. However, it is evident that HE students have changing educational expectations and requirements (Shaw and Fairhurst 2008). Congruent with literature which suggests that students want greater control over their learning experiences (Carlson 2005; Feiertag and Berge 2008; Stewart 2009), students believed they should be given greater control over what is taught and how—19.80% (*n* = 20) respondents ‘agreed strongly’ and 45.54% (*n* = 46) ‘agreed somewhat’. Finney and Finney (2010) suggest students who view themselves as consumers are more likely to see themselves as entitled to receive positive academic results. Yet when asked to what extent respondents agreed with the following statement—‘My grade should always be bumped up if I’m close to a grade boundary’—results were varied: 8.91% (*n* = 9) respondents ‘agreed strongly’, 20.79% (*n* = 21) ‘agreed somewhat’, 29.70% (*n* = 30) ‘neither agreed nor disagreed’ and 19.80% (*n* = 20) ‘disagreed strongly’.

- ‘Do you think your grade should be bumped up if you’re close to a grade boundary?’
  o ‘Yes, only if it’s within one mark of the grade boundary. I think we should always be rewarded for the effort and work that’s gone into something, and at the end of the day that 1% could just be due to bias marking. You should at least have the chance to argue your case’ (Participant C, Female, UG, Geography, 6 contact hours a week)

For respondents who recognised the shifting role of students within a marketised system, several core themes and concerns were apparent. A general theme amongst all respondents was the concern surrounding ‘value for money’, which consistent with findings from Tomlinson (2017) was accompanied by a longing for greater transparency over resources provided to them through fee revenue:

- ‘Do you feel that you receive value for money?’
- ‘Yes, I do in the long run, but in the short run I sometimes feel that if every course is worth £9000, when for maths we don’t have a lot of high tech stuff, we just have lectures, I do feel like where does some of that money go? When I’m just paying for lecturers to come up and say stuff”. (Participant I, Male, UG, Mathematics, 16 contact hours a week)

Several respondents identified a direct correlation between value for money and the number of contact hours and resources provided to them:

- ‘Do you think the payment of tuition fees ensures you a higher level of education than if it were free?’
- ‘Yes—the standard of facilities will go through the roof. Tuition fees have funded three months of fieldwork and excellent dedicated lab space’. (Participant J, Male, PG, Earth Sciences, 20 contact hours a week)

Another participant who received significantly fewer contact hours a week, was less convinced:

- ‘Yes, students demand more of the services offered to them, but so they should. I often think, ‘where is my £9000 going?’, when lecturers don’t reply to emails, and aren’t in their office hours. I often think, why are they not there?’ (Participant C, Female, UG, Drama, 6 contact hours a week)

Respondents who resisted a ‘consumer’ identity still identified they should be treated ‘in a certain way’:

- ‘Do you see yourself as a consumer or customer of your university?’
  o ‘No, I’d class myself as a student. I think it’s very different. I don’t think unis are customer facing environments. I think lecturers should treat you in a certain way, but not in the same way you treat a customer in a shop’. (Participant H, Female, PG, Management/Law, 6 contact hours a week)

Students highlighted the need to remain active in achieving their desired goals and outcomes, identifying ‘you have to work for it’ (Participant H), thus contesting a body of literature which states that students increasingly wish to put forward little effort (Sacks 1996; Trout 1997):

o ‘You’ve got to put the work in. Technically we are the customer because we are paying for it. But you also have to invest in other ways, in work, energy and effort. Otherwise you’re wasting your own money.’ (Participant C, Female, UG, Drama, 6 contact hours a week)

The findings of this research support the view that paying money in exchange for a service naturally results in feelings of entitlement (Finney and Finney 2010). Yet supporting findings by Budd (2016), there is little evidence of the ‘deflection of responsibility’ that White (2007) describes in their research on students’ experiences of the commodification of HE sector in Australia. Whilst students’ express feelings of greater entitlement due to the payment of tuition fees, they do not perceive achieving a degree as their ‘right’. They do, however, feel entitled to greater contact hours, frequent access to staff, with expectations they ‘respond in good time’ (Participant E) (supporting findings from Bunce et al. 2016), and high-class facilities. Arguably, this could assist students in achieving more positive academic results. Whilst students do not feel entitled to the guarantee of a degree due to a financial exchange, they do feel entitled to the tools which may assist them on their way in doing so.

## Conclusion

Through a critical interpretation of the attitudes, expectations, behaviours, and relationships held by students regarding HE, this paper challenges the student-consumer ideology and offers more nuanced interpretations of student identities in a marketised British HE sector. It provides a theorisation that attests to the complexity, diversity, and individuality of HE student identities, supporting that there is not a ‘one size fits all’ approach which can be used to describe or define HE students. Significantly, the research highlights the problematic position of the student-consumer in policy and media discourse.

Empirically, the paper contributes to a small, but growing, body of literature which considers the perspectives of students themselves when positioning students as consumers. Through a cross-national and cross-institutional study, this research has cemented the perspective of British HE students in existing literature. This research finds that consumerist discourses are present within student identities, largely due to the payment of tuition fees, and are in part framing students’ expectations and relationship with HE. A stronger consumer identity was held by those who made a direct financial exchange (for example, if they paid for their tuition fees directly from their bank accounts, rather than via the student loan process). Furthermore, a general theme amongst all respondents was the concern surrounding ‘value for money’. Several respondents identified a direct correlation between value for money and the number of contact hours and resources provided to them. Yet despite some students identifying as consumers, and others who whilst not self-identifying directly as consumers expressing consumerist behaviours (such as perceiving lecturers as providing ‘customer support’), students challenged that they are passive consumers. Students did not believe they should be guaranteed a degree due to the payment of tuition fees. Instead, they recognised the need to remain active co-producers in their educational outcomes, foregrounding the importance of learning and hard work over more instrumental concerns typically implied within policy (Brooks and Abrahams 2020). The findings of this paper support current literature as to why students attend university, with the overriding motivation for studying at HE level being to advance employability. However, this was not at the expense of students’ willingness to engage and excel as scholars. Whilst those who pay for commodities or services in other walks of life would logically perceive themselves as consumers, students identified that their engagement with HE differs in the degree of participation in the relationship between service provider and consumer. The passive nature of buying a coffee in a café and waiting whilst the coffee is produced and transferred to the consumer by the barista is far different from the relationship between students and HE, and this is recognised by students in this research.

This research critiques the view that the student-consumer is the dominant identity of HE students. These findings have practical implications for how students and their relationship to HE should be understood in policy terms. Policymakers should be cautious when attempting to measure the ‘quality’ of HE simply in terms of employment outcomes, which overlooks the role of HE in the personal development of students and the development of students into critical scholars, who seek to learn and engage with intellectual content. By positioning students solely as consumers, this oversimplifies their roles and activities within HE. However, this research also has important implications for how HE policy should manage student expectations around their relationship to HE, particularly so in a challenging COVID-19 context. This is particularly the case with the ongoing debate about ‘value for money’ and the shift from in-person to online teaching, for example.

Debates regarding the identity of the student-consumer are likely to intensify due to the impacts of Brexit and COVID-19 on HE internationally. The UK’s announcement to end home fee status for new EU students studying in the UK from 2021 has likely further exaggerated the marketisation of HEIs as efforts to attract high fee-paying students intensify. Furthermore, whilst this research was conducted prior to the impact of COVID-19 on the HE sector, significant changes to the HE sector, including a transition to online teaching, is likely to fuel further debate around the value of HE. How then will student identities be impacted in an international post-pandemic HE system? The arguments presented within this paper are also relevant to an international audience, as the marketisation and neoliberalisation of HE is an increasingly global phenomenon. In further exploring the impacts of the marketisation of HE on student identities, future research should be encouraged to explore how attitudes of consumerism, entitlement, and instrumentalism differ across the devolved administrations of England, Scotland, and Wales, given differences in HE policy, as well as across institutional type, subject, gender, and age, for example. As the internationalisation of HE continues to gain pace, differences in how domestic versus international students perceive HE are also important to consider. These constitute important areas of enquiry if the impact of marketisation on HE students’ identities is to be fully understood.

## Data Availability

The data that support the findings of this research are available from the author upon reasonable request.

## References

[CR1] Barnett R, Molesworth M, Scullion R, Nixon E (2011). The marketised university: defending the indefensible. The Marketisation of Higher Education and the Student as Consumer.

[CR2] Bossick MJ (2009). Student consumerism in higher education: an analysis of relationships with institutions.

[CR3] Braun V, Clarke V (2006). Using thematic analysis in psychology. Qual Res Psychol.

[CR4] Brooks R (2021). Students as consumers? The perspectives of students' union leaders across Europe. Higher Educ Q.

[CR5] Brooks R, Abrahams J (2020). European higher education students: contested constructions. Sociol Res Online.

[CR6] Brooks R, Gupta A, Jayadeva S, Abrahams J (2021). Students’ views about the purpose of higher education: a comparative analysis of six European countries. High Educ Res Dev.

[CR7] Brown R, Brown R (2011). Introduction. Higher education and the market.

[CR8] Brown R (2015). The marketisation of higher education: issues and ironies.

[CR9] Budd R (2016). Undergraduate orientations towards higher education in Germany and England: problematizing the notion of ‘student as customer’. High Educ.

[CR10] Bunce L, Baird A, Jones SE (2016). The student-as-consumer approach in higher education and its effects on academic performance. Stud High Educ.

[CR11] Carlson S (2005) The Net generation goes to college. *The Chronicle of Higher Education,* 7 October. http://www.chronicle.com/article/The-Net-Generation-Goes-to/12307. Accessed 11 Mar 2021

[CR12] Cheal DJ (1979). Hegemony, ideology, and contradictory consciousness. Sociol Q.

[CR13] Ciani KD, Summers JJ, Easter MA (2008). Gender differences in academic entitlement among college students. J Genet Psychol.

[CR14] Clayson DE, Hayley DA (2005). Marketing models in education: students as customers, products or partners. Mark Educ Rev.

[CR15] Consumer Rights Act (2015) https://www.legislation.gov.uk/ukpga/2015/15/contents/enacted. Accessed 11 Mar 2021

[CR17] Cuthbert R (2010). Students as customers?. High Educ Rev.

[CR18] Davies P, Mangan J, Hughes A (2013). Labour market motivation and undergraduates’ choice of degree subject. UK Educ Res J.

[CR19] Dearing R (1997). Higher education in the learning society: report of the national committee of inquiry into higher education.

[CR20] Delucchi M, Korgen G (2002). “We’re the customer—we pay the tuition”: student consumerism among undergraduate sociology majors. Teach Sociol.

[CR21] Feiertag J, Berge ZL (2008). Training generation N: how educators should approach the Net generation. Educ Train.

[CR22] Finney T, Finney R (2010). Are students their Universities’ customers? An exploratory study?. Educ Train.

[CR23] Foskett N, Molesworth M, Scullion R, Nixon E (2010). Markets, government, funding and the marketisation of UK higher education. The marketisation of higher education and the student as consumer.

[CR24] Foster JD, Campbell WK, Twenge JM (2003). Individual differences in narcissism: inflated self-views across the lifespan and around the world. J Res Pers.

[CR25] Fromm E (1976). To have or to be?.

[CR30] Government of the United Kingdom (2017) Higher Education and Research Act 2017 [Online]. https://www.legislation.gov.uk/ukpga/2017/29/contents/enacted. Accessed 11 Feb 2022

[CR27] Gramsci A (1971). Selections from the prison notebooks.

[CR28] Greenberger E, Lessard J, Chen C (2008). Self-entitled college students: contributions of personality, parenting and motivational factors. J Youth Adolesc.

[CR29] Hemsley-Brown J, Oplatka I (2015). University choice: what do we know, what don’t we know and what do we still need to find out?. Int J Educ Manage.

[CR31] Hirschman AO (1970). Exit, voice, and loyalty.

[CR32] Hoover E (2007). Here’s you looking at you, kid: study says many students are narcissists. Chron Higher Educ.

[CR33] Jayadeva S, Brooks R, Gupta A, Abrahams J, Lažetič P, Lainio A (2021). Are Spanish students customers? Paradoxical perceptions of the impact of marketisation on higher education in Spain. Sociol Res Online.

[CR34] Jongbloed B (2003). Marketisation in higher education, Clark’s triangle and the essential ingredients of markets. High Educ Q.

[CR35] Kalafatis S, Ledden L (2013). Carry-over effects in perceptions of educational value. Stud High Educ.

[CR36] Kaye T, Bickel R, Birtwistle T (2006). Criticizing the image of the student as consumer: examining legal trends and administrative responses in the US and UK. Educ Law.

[CR37] King K (1993). Education policy in a climate of entitlement: the South African case. Perspect Educ.

[CR38] Labaree D (1997). Public good, private goods: the american struggle over education goal. Am Educ Res J.

[CR39] Lane L, Dent S, Lane L, Strike T (2017). Introduction: students as co-creators or consumers?. Collaboration, communities and competition: International Perspectives from the Academy.

[CR40] Lomas L (2007). Are students customers? Perceptions of academic staff. Qual High Educ.

[CR41] Marginson S (2006). Investment in the self: the government of student financing in Australia. Stud High Educ.

[CR42] Mawji S (2016) The consumer rights act and higher education. https://www.matchsolicitors.com/news/2016-11-02-the-consumer-rights-act-and-higher-education. Accessed 11 Mar 2021

[CR43] McCulloch A (2009). The student as co-producer: learning from public administration about the Student-University relationship. Stud High Educ.

[CR44] McCollough MA, Gremler DD (1999). Guaranteeing student satisfaction: an exercise in treating students as customers. J Mark Educ.

[CR45] Molesworth M, Nixon E, Scullion R (2009). Having, being and higher education: the marketisation of the university and the transformation of the student into consumer. Teach High Educ.

[CR46] Morrison A (2016). The responsibilized consumer: neoliberalism and english higher education policy. Cult Stud Crit Methodol.

[CR47] Morrow W (1994). Entitlement and achievement in education. Stud Philos Educ.

[CR48] Muddiman E (2018). Instrumentalism amongst students: a cross-national comparison of the significance of subject choice. UK J Sociol Educ.

[CR49] Naidoo R, Jamieson I (2005). Empowering participants or corroding learning? Towards a research agenda on the impact of student consumerism in higher education. J Educ Policy.

[CR50] Naidoo R, Shankar A, Veer E (2011). The consumerist turn in higher education: policy aspirations and outcomes. J Mark Manage.

[CR51] Neary M, Winn J, Bell L, Stevenson H, Neary M (2009). The student as producer: reinventing the student experience in higher education. The future of higher education: policy, pedagogy and the student experience.

[CR52] Nixon E, Scullion R, Hearn R (2016). Her majesty the student: marketised higher education and the narcissitic (dis) satisfactions of the Student-Consumer. Stud High Educ.

[CR53] Ng ICL, Forbes J (2009). Education as service: the understandings of University experience through the service logic. J Mark High Educ.

[CR54] Obermiller C, Fleenor P, Raven P (2005). Students as customers or products: perceptions of faculty and students. Mark Educ Rev.

[CR55] Office for Students (2022) National Student Survey—NSS. https://www.officeforstudents.org.uk/advice-and-guidance/student-information-and-data/national-student-survey-nss/. Accessed 18 Jan 2022

[CR56] Sacks P (1996). Generation X goes to college: an eye opening account of teaching in postmodern America.

[CR57] Saldaña J (2016). The coding manual for qualitative researchers.

[CR58] Saunders DB (2015). They do not buy it: exploring the extent to which entering first-year students view themselves as customers. J Mark High Educ.

[CR59] Saunders M, Lewis P, Thornhill A (2012). Research methods for business students.

[CR60] Schwartzman R (2013). Consequences of commodifying education. Acad Exchange Q.

[CR61] Sharif AM (2014) Students as Prosumers. https://universitybusiness.co.uk/Article/students_as_prosumers/. Accessed 11 Mar 2021

[CR62] Shankar A, Fitchett J (2002). Having, being and consumption. J Mark Manage.

[CR63] Shaw S, Fairhurst D (2008). Engaging a new generation of graduates. Educ Train.

[CR64] Silverio SA, Wilkinson C, Wilkinson S (2021). The powerful student consumer and the commodified academic: a depiction of the marketised UK higher education system through a textual analysis of the ITV drama cheat. Sociol Res Online.

[CR65] Singleton-Jackson JA, Jackson DL, Reinhardt J (2010). Students as consumers of knowledge: are they buying what we’re selling?. Innov High Educ.

[CR66] Stewart K (2009). Lessons from teaching millennials. Coll Teach.

[CR67] Svensson G, Wood G (2007). Are university students really customers? When illusion may lead to delusion for all!. Int J Educ Manage.

[CR68] Tavares O, Cardoso S (2013). Enrolment choices in Portuguese higher education: do students behave as rational consumers?. High Educ.

[CR69] Telling K (2018). Selling the liberal arts degree in England: unique students, generic skills and mass higher education. Sociology.

[CR70] Tight M (2013). Students: customers, clients or pawns?. High Educ Policy.

[CR71] Tomlinson M (2017). Student perceptions of themselves as ‘consumers’ of higher education. UK J Sociol Educ.

[CR72] Trout PA (1997). What the numbers mean: providing a context for numerical student evaluations of courses. Change Mag Higher Educ Learn.

[CR73] UCAS (2022) Postgraduate fees and funding. https://www.ucas.com/postgraduate/postgraduate-fees-and-funding. Accessed 02 Mar 2022

[CR74] University of Edinburgh (2022) How much will it cost? https://www.ed.ac.uk/studying/undergraduate/fees-finance/scotland/cost. Accessed 28 Feb 2022

[CR75] von Alberti-Alhtaybat L, Abdelrahman N, Al-Htaybat K (2017). The effect of different higher education fee policies on education: a comparison between England and Germany. Int J Public Sect Manage.

[CR76] White NR (2007). “The customer is always right?”: student discourse about higher education in Australia. High Educ.

[CR77] Williams J (2013). Consuming higher education: why learning can’t be bought.

[CR78] Wright K (2005). Researching Internet-based populations: advantages and disadvantages of online survey research, online questionnaire authoring software 68 packages, and web survey services. J Comput-Mediat Commun.

[CR79] Zandstra R, Dunne E (2009) Students as change agents: new ways of engaging with learning and teaching in Higher Education. http://escalate.ac.uk/8242. Accessed 31 Mar 2021

